# Supplementary material for: A scalable membrane electrode assembly architecture for efficient electrochemical conversion of CO2 to formic acid
Source: Nat Commun. 2023 Nov 22;14:7605. doi: 10.1038/s41467-023-43409-6 (PMC10663610; doi:10.1038/s41467-023-43409-6)
Supplement: Supplementary file 1 — Supplementary Information [file 41467_2023_43409_MOESM1_ESM.pdf]

Supporting Information

# A Scalable Membrane Electrode Assembly Architecture for Efficient Electrochemical Conversion of $CO_2$ to Formic Acid

Leiming Hu[1], Jacob A. Wrubel[1], Carlos Baez-Cotto[2], Fry Intia[1], Jae Hyung Park[3], Arthur Jeremy Kropf[3], Nancy Kariuki[3], Zhe Huang[4], Ahmed Farghaly[3], Lynda Amichi[5], Prantik Saha[1], Ling Tao[4], David A. Cullen[5], Deborah J. Myers[3], Magali S. Ferrandon[3], KC Neyerlin[1,*].

1. Chemistry and Nanoscience Center, National Renewable Energy Laboratory, Golden, CO, USA
2. Materials Science Center, National Renewable Energy Laboratory, Golden, CO, USA
3. Chemical Sciences and Engineering Division, Argonne National Laboratory, Lemont, IL, USA
4. Catalytic Carbon Transformation & Scale-Up Center, National Renewable Energy Laboratory, Golden, CO, USA
5. Center for Nanophase Materials Sciences, Oak Ridge National Laboratory, Oak Ridge, TN, USA

*Corresponding author: Kenneth.Neyerlin@nrel.gov

Table S 1. Summary of $CO_2$ to formate/formic acid cell configurations and performance

| Configuration | GDE active area [cm2] | Cathode catalyst | Cell voltage [V] | Current density ($A/cm^2$) | Formate/Formic acid FE [%] | Reference |
|---|---|---|---|---|---|---|
| PCEM/AEM composite | 25 | Bi2O3 | 1.06 | 25.0 | 29.40 | This work |
| | 25 | | 1.18 | 50.0 | 37.90 | |
| | 25 | | 1.49 | 75.0 | 45.97 | |
| | 25 | | 1.62 | 100.0 | 48.97 | |
| | 25 | | 1.68 | 125.0 | 60.20 | |
| | 25 | | 1.73 | 150.0 | 66.67 | |
| | 25 | | 1.85 | 200.0 | 70.92 | |
| | 25 | | 1.92 | 250.0 | 78.04 | |
| | 25 | | 1.94 | 300.0 | 78.64 | |
| | 25 | | 2.05 | 400.0 | 78.57 | |
| | 25 | | 2.27 | 500.0 | 71.02 | |
| Catholyte Config | 80 | Sn plate | 3.50 | 30.0 | 80.00 | [19] |
| Catholyte Config | 2.55 | SnO2 NP | 2.47 | 400.0 | 72.00 | [21] |
| Catholyte Config | 10 | SnO2 NP | 4.00 | 200.0 | 54.00 | [22] |
| Catholyte Config | 10 | Sn plate | 2.80 | 12.0 | 71.40 | [23] |
| Catholyte Config | 2.7 | SnO2 NP | N/A | 500.0 | 70.00 | [24] |
| Catholyte Config | 4 | Sn NP | 5.40 | 100.0 | 16.00 | [25] |
| Catholyte Config | 25 | SnO2 | 2.58 | 10.0 | 59.76 | [34] |
| | 25 | | 2.81 | 20.0 | 60.46 | |
| | 25 | | 2.87 | 30.0 | 64.17 | |
| | 25 | | 3.00 | 40.0 | 65.79 | |
| | 25 | | 3.12 | 50.0 | 69.28 | |
| | 25 | | 3.19 | 60.0 | 72.06 | |
| | 25 | | 3.35 | 80.0 | 72.52 | |
| | 25 | | 3.50 | 100.0 | 74.37 | |
| | 25 | | 3.65 | 125.0 | 79.01 | |
| | 25 | | 3.84 | 150.0 | 84.12 | |
| | 25 | | 3.96 | 175.0 | 87.36 | |
| | 25 | | 4.15 | 200.0 | 91.53 | |
| | 25 | | 4.46 | 250.0 | 86.40 | |
| | 25 | | 4.71 | 300.0 | 91.02 | |
| | 25 | | 5.46 | 400.0 | 90.74 | |
| | 25 | | 6.05 | 500.0 | 88.60 | |
| Catholyte Config | 2.75 | | 3.10 | 98.2 | 94.08 | [26] |
| | 2.75 | | 3.39 | 178.8 | 90.34 | |
| | 2.75 | | 3.70 | 277.7 | 87.85 | |
| | 2.75 | | 4.00 | 380.9 | 89.72 | |
| Catholyte Config | 10 | Bi np | 3.10 | 90.0 | 92.40 | [27] |
| | 10 | | 3.70 | 150.0 | 83.10 | |
| | 10 | | 4.20 | 200.0 | 80.40 | |
| | 10 | | 5.40 | 300.0 | 70.60 | |
| | 10 | | 3.10 | 90.0 | 89.50 | |
| | 10 | | 4.30 | 200.0 | 62.70 | |
| Single Membrane Config | 25 | Sn np | 1.90 | 23.4 | 80.54 | [29] |
| | 25 | | 2.00 | 32.2 | 87.88 | |
| | 25 | | 2.10 | 44.4 | 94.99 | |
| | 25 | | 2.20 | 56.1 | 92.70 | |
| | 25 | | 2.30 | 68.1 | 86.27 | |

| | | | | | | |
|---|---|---|---|---|---|---|
| | 25 | | 2.40 | 79.2 | 76.41 | |
| | 25 | | 2.50 | 93.2 | 60.11 | |
| Interlayer Config | 4 | 2D-Bi | 2.57 | 2.7 | 53.73 | [30] |
| | 4 | | 2.74 | 6.9 | 71.39 | |
| | 4 | | 2.89 | 11.2 | 79.60 | |
| | 4 | | 3.00 | 21.3 | 87.31 | |
| | 4 | | 3.08 | 34.5 | 92.54 | |
| | 4 | | 3.14 | 50.1 | 88.56 | |
| | 4 | | 3.18 | 67.2 | 89.55 | |
| | 4 | | 3.22 | 84.6 | 85.57 | |
| | 4 | | 3.27 | 101.0 | 81.84 | |
| Interlayer Config | 4 | 2D-Bi | 1.97 | 1.3 | 46.41 | [30] |
| | 4 | | 2.25 | 5.1 | 62.87 | |
| | 4 | | 2.36 | 12.1 | 77.25 | |
| | 4 | | 2.41 | 19.7 | 91.92 | |
| | 4 | | 2.52 | 36.9 | 95.51 | |
| | 4 | | 2.61 | 62.4 | 92.51 | |
| | 4 | | 2.68 | 100.0 | 91.62 | |
| | 4 | | 2.76 | 150.3 | 88.62 | |
| | 4 | | 2.81 | 200.6 | 82.93 | |
| Interlayer Config | 5 | | 3.23 | 100.0 | 68.00 | [31] |
| | 5 | | 3.52 | 200.0 | 74.00 | |
| | 5 | | 3.76 | 250.0 | 73.00 | |
| Interlayer Config | 1 | | 1.35 | 50 | 50 | [15] |
| | 1 | | 1.39 | 100 | 100 | |
| | 1 | | 1.44 | 150 | 150 | |
| | 1 | | 1.49 | 200 | 200 | |
| | 1 | | 1.75 | 300 | 300 | |
| Single CEM configuration | Cell area ($cm^2$) | Catalyst | | Current density | Energy consumption (kWh/kmol) | [28] |
| | 10 | Bi | | 45 | 207.4 | |
| | 10 | | | 100 | 409.8 | |
| | 10 | | | 200 | 546.0 | |

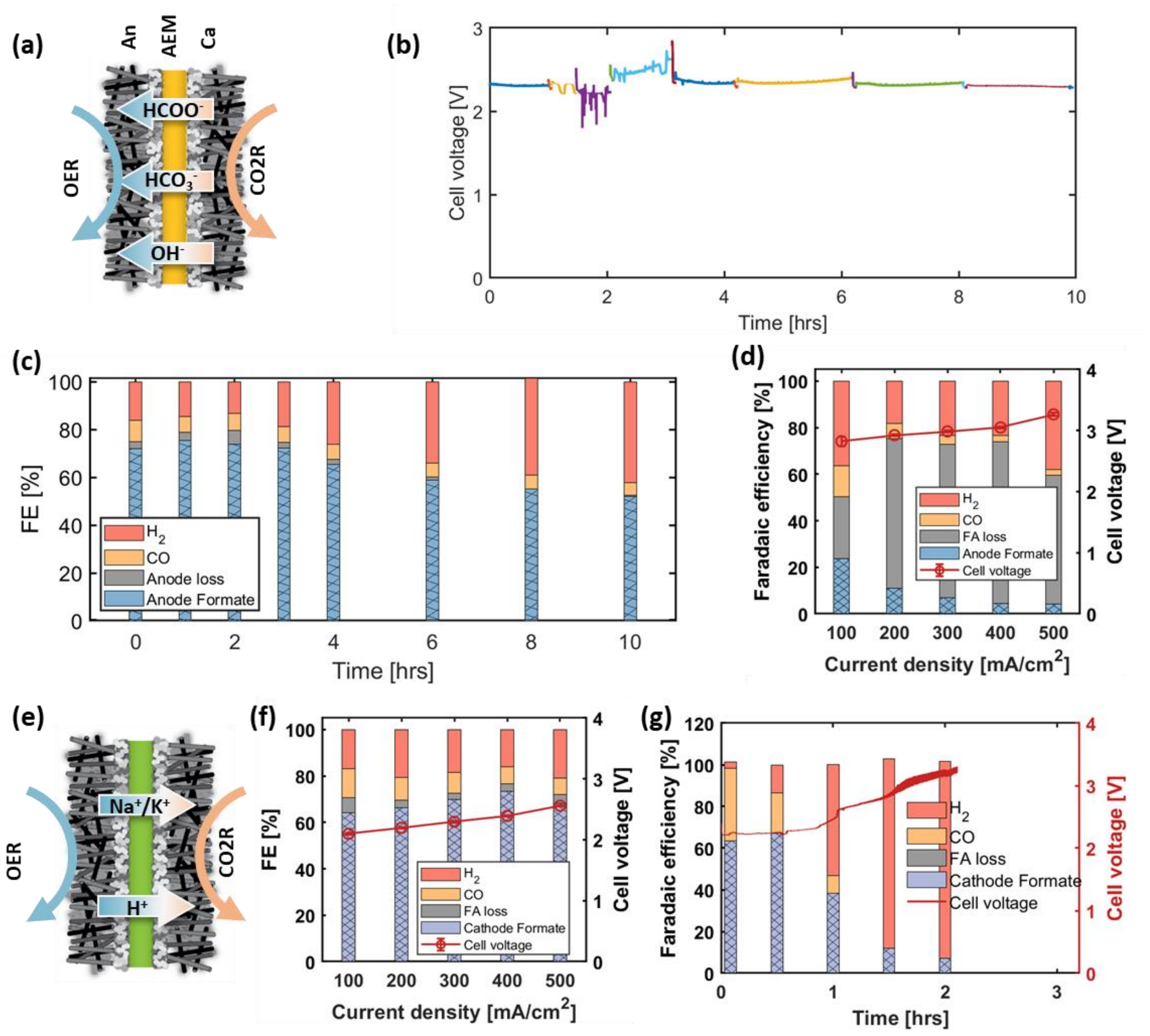


Figure S1. (a) Schematic of the zero-gap MEA utilizing a single AEM membrane, with CO2R at the cathode with the Oxygen evolution reaction at the anode. (b) Cell voltage and (c) FE vs. time at 200 mA/cm$^2$ with 1M KOH used at the anode. (d) FE and cell voltage at different current densities when 0.1 M KOH is used at the anode. (e) Schematic of the zero-gap MEA utilizing a single CEM membrane, with CO2R at the cathode with the Oxygen evolution reaction at the anode. (f) FE and cell voltage at different current densities when 0.1 M KOH is used at the anode. (g) Cell voltage and

FE vs. time at 200 mA/cm$^2$ with 1M KOH used at the anode. Error bars stand for S.D. from three different measurements.

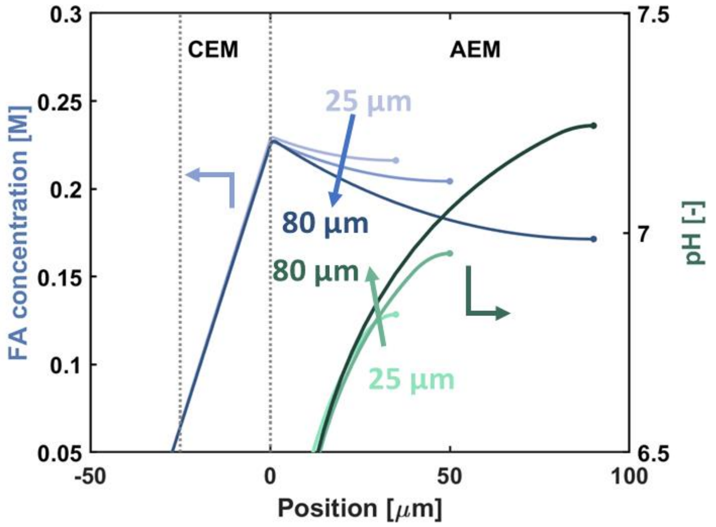


Figure S 2. Simulation results of formic acid concentration and pH across the CEM/AEM interface with different AEM thicknesses.

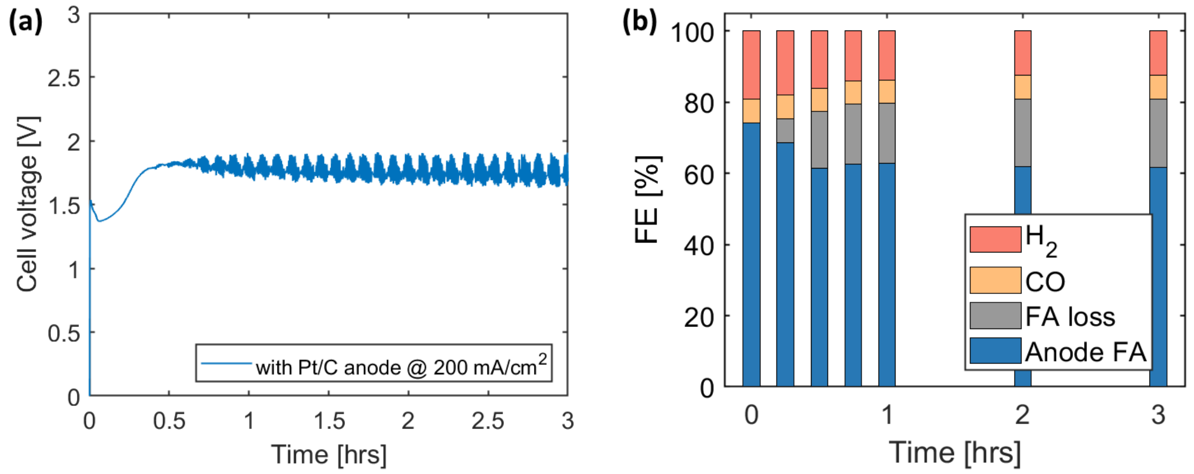


Figure S3. Durability of the system with an 80 mm AEM and perforated CEM at 60ºC. (a) First 3 hours of cell voltage vs. time at 200 mA/cm$^2$ (b) First 3 hours of FE vs. time at 200 mA/cm$^2$.

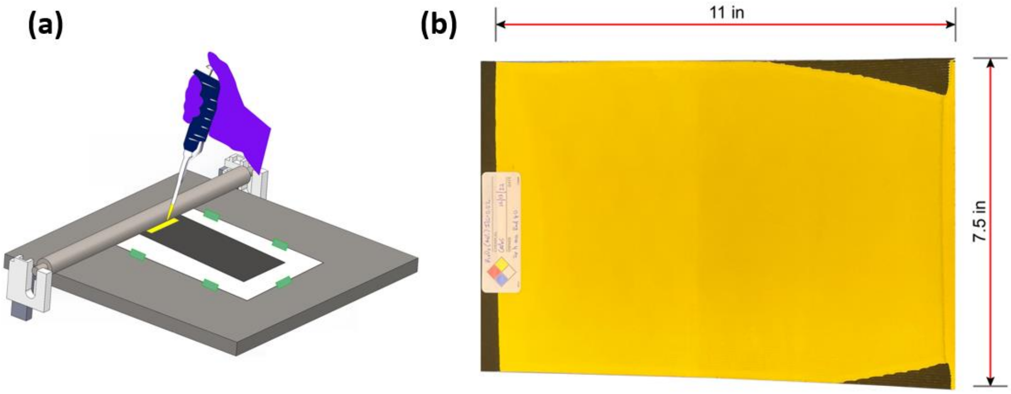


Figure S4 (a) Illustration of the rod coating process. (b) Photo of the rod coated $Bi_2O_3$ gas diffusion electrode.

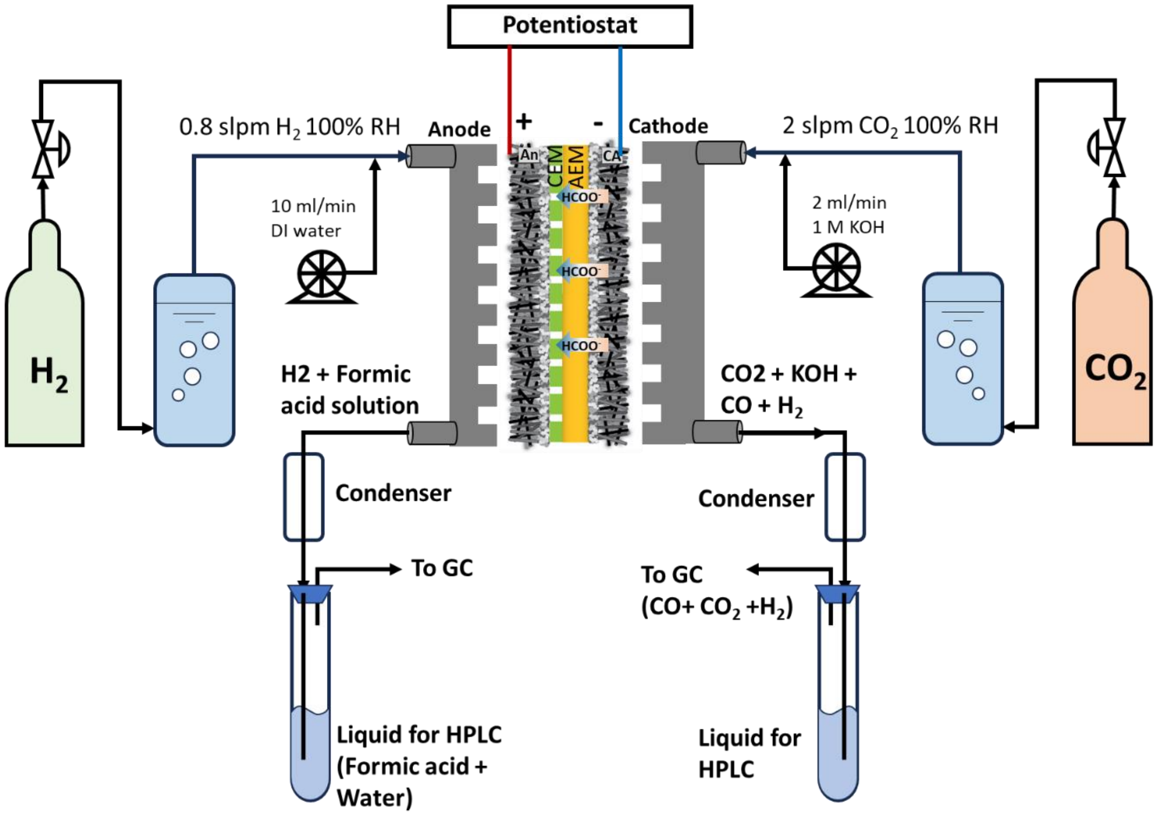


Figure S5. Detailed information about all the inputs and outputs of the cell during electrochemical cell testing. The anode is supplied with a mixture of 0.8 slpm $H_2$ at 100% RH and 10 mL/min DI water. The cathode is supplied with 2 slpm 100% $CO_2$ gas mixed with 2 mL/min 1 molar KOH. Formic acid is collected at the anode, and the CO and $H_2$ are collected from the cathode.

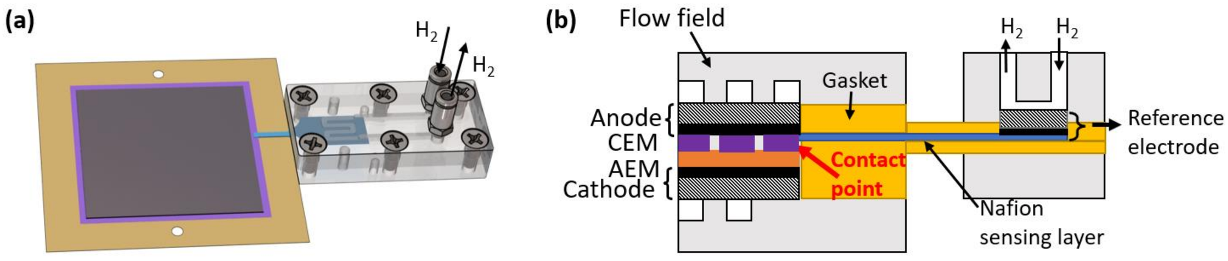


Figure S 6. (a) Schematic of the reference electrode hardware and the Nafion sensing tip. (b) Cross-sectional image of the reference electrode and the MEA.

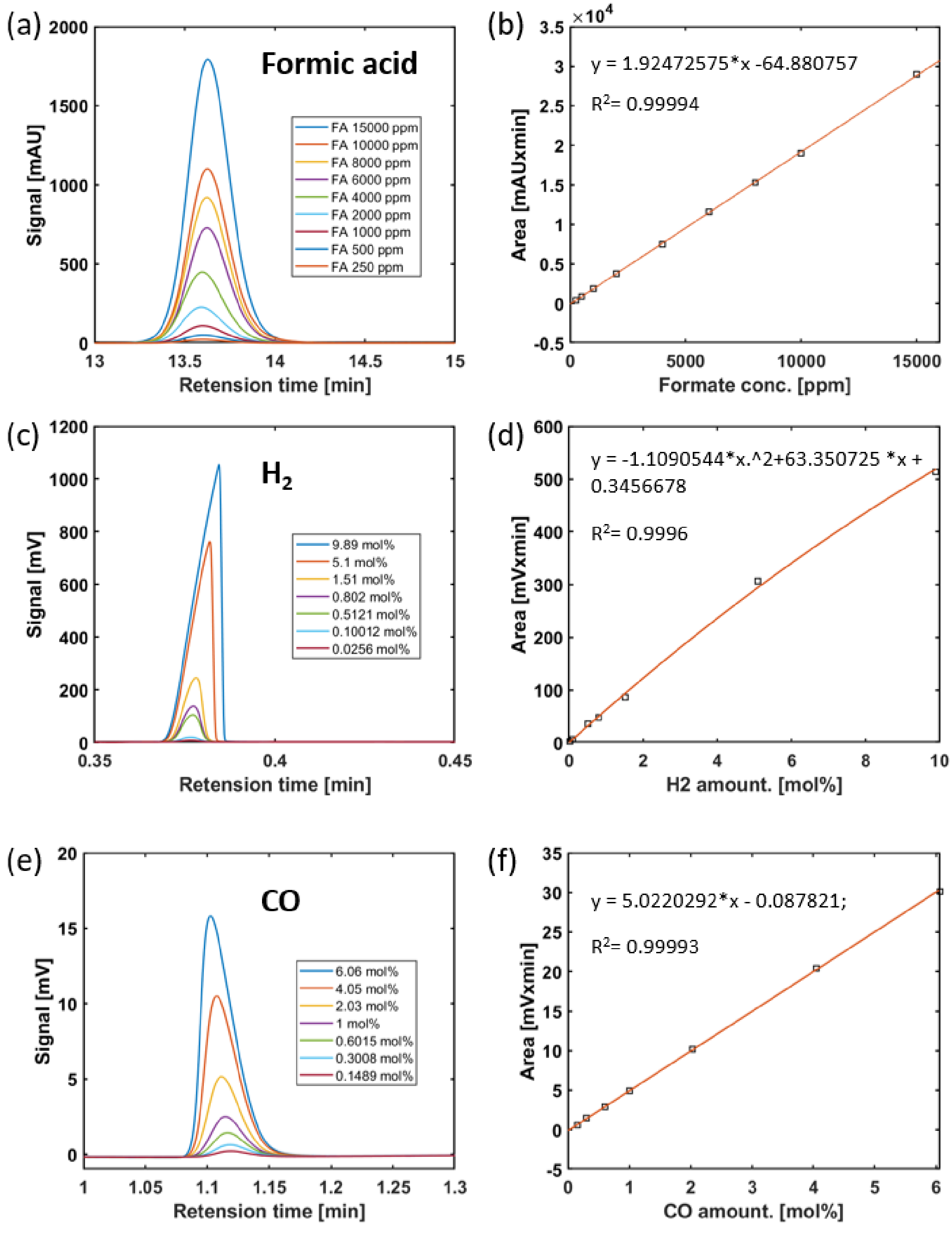


Figure S 7. (a) HPLC chromatogram for formic acid standards from 250 ppm to 15000 ppm and (b) the calibration curve for formic acid. GC chromatogram and calibration curve for $H_2$(c, d) and CO (e, f).

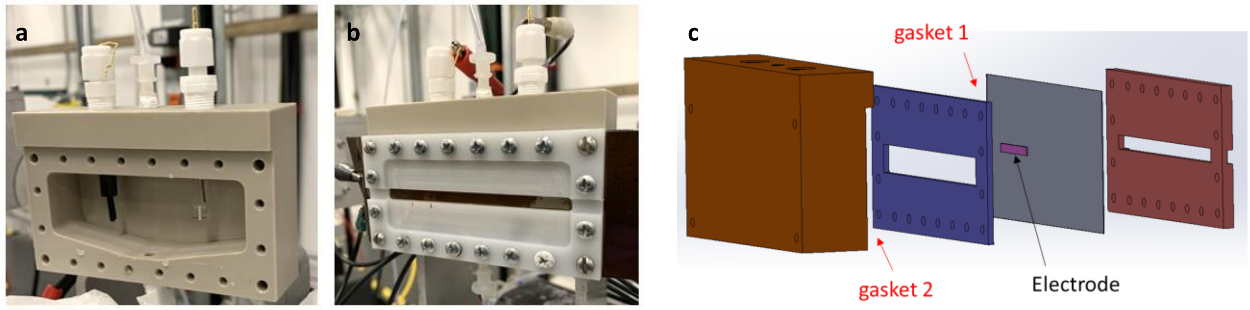


Figure S 8. In-situ XAFS cell, picture of the main body (a), picture of the mounted cell with windows and electrodes (b), and schematic of the cell components (c).

## Model Theory

As noted in the main text, the model used in this work is a direct extension of one published previously[1]. A schematic of the model geometry is shown below in Figure S 9, along with a general sketch of the species transport motifs and space charge regions (SCRs). SCRs arise in the vicinity of an ion exchange membrane (e.g., CEM|Pore and AEM|Pore), or at the interface between two ion exchange membranes with different fixed charge concentrations (e.g., CEM|AEM).

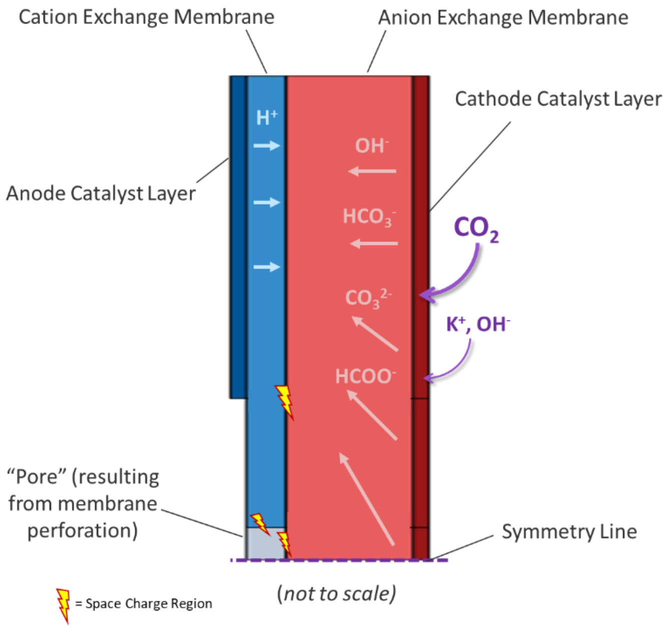

Figure S9. Schematic of the 2D geometry used for the Poisson Nernst Planck Transport model.

The flux, $J_k$, of aqueous species is modeled by the Nernst Planck equation,

$$J_k = -D_k\left(\nabla c_k + \frac{z_k F}{RT} c_k \nabla \phi_I\right) \quad (1)$$

where *k* denotes the *k*$^{th}$ aqueous species, *D* is the diffusivity, *c* is the concentration, $z_k$ is the valence, and $\phi_I$ is the electrostatic potential in the ion-conducting phase. The electrostatic potential is obtained from the Poisson equation for electrostatics,

$$\nabla^2 \phi_I = -\frac{\rho}{\varepsilon} = -\frac{F}{\varepsilon}\sum_k z_k c_k \quad (2)$$

where $\rho$ is the space charge density, and $\varepsilon$ is the permittivity. The space charge density can also be represented as

$$\rho = F\sum_k z_{fix} c_{fix} + z_k c_k \quad (3)$$

where the subscript 'fix' refers to the concentration fixed charge groups: 1.1 M for the CEM, 1.5 M for the AEM, and 0 M for the pore. The governing species equation is obtained by applying continuity to Eq. (1), and including chemical ( $\dot{R}_{chem}$ ) and electrochemical ($\dot{R}_{echem}$) reaction source terms:

$$\frac{\partial c_k}{\partial t} = \nabla \cdot \left(D_k\left(\nabla c_k + \frac{z_k F}{RT} c_k \nabla \phi_I\right)\right) + \dot{R}_{chem} + \dot{R}_{echem} \quad (4)$$

The reaction source terms are identical to those used in[1], and complete expressions for them can be found in the SI of that paper.

Charge conservation in the electronic conducting phases is expressed according to Eq. 5,

$$\nabla \cdot i_e = -\dot{R}_{gen} \quad (5)$$

where the electronic current density, $i_e$, is governed by Ohm's law

$$i_e = \sigma_{eff} \nabla \phi_e \quad (6)$$

and $\sigma_{eff}$ is the effective conductivity, $\phi_e$ is the potential in the electronic phase ($\phi_A$ for anode and $\phi_C$ for cathode), and $\dot{R}_{gen}$ is a volumetric charge generation term reflecting the electrochemical reactions, similar to $\dot{R}_{echem}$ in Eq. 4.

The numerous chemical and electrochemical reactions, combined with the three distinct SCRs, result in a numerically stiff system of equations. To solve this system, an initialization procedure was developed to make the system more tractable. First, a damping parameter "$\delta$" was introduced in front of the source terms, as seen in Eq. 7.

$$\frac{\partial c_k}{\partial t} = \nabla \cdot \left( D_k \left( \nabla c_k + \frac{z_k F}{RT} c_k \nabla \phi_I \right) \right) + \delta \dot{R}_{chem} \quad (7)$$

The system was solved from $t = 0 - 60s$ for $\delta = 10^{-5} \rightarrow 10^{-4} \rightarrow 10^{-3} \rightarrow 10^{-2} \rightarrow 10^{-1} \rightarrow 10^{0}$, and the solution of each step was used as the initial condition for the next. The electrochemical reactions were set to zero and the electrode potentials were not solved for. This procedure slowly introduced the effects of the chemical reactions and reduced the complications associated with large ionic currents. Note that for $\delta = 1$ the full effect of the chemical reactions is represented. Next, the electrode potentials were initialized by solving only Eq. 5 (for both anode and cathode), using the final solution of Eq. 7 ($\delta = 1$). Lastly, the fully coupled system was solved for all current densities of interest, generally starting at 1 mA/cm$^2$ and always using the solution from the previous step as initial conditions for the next.

**Temperature effect**

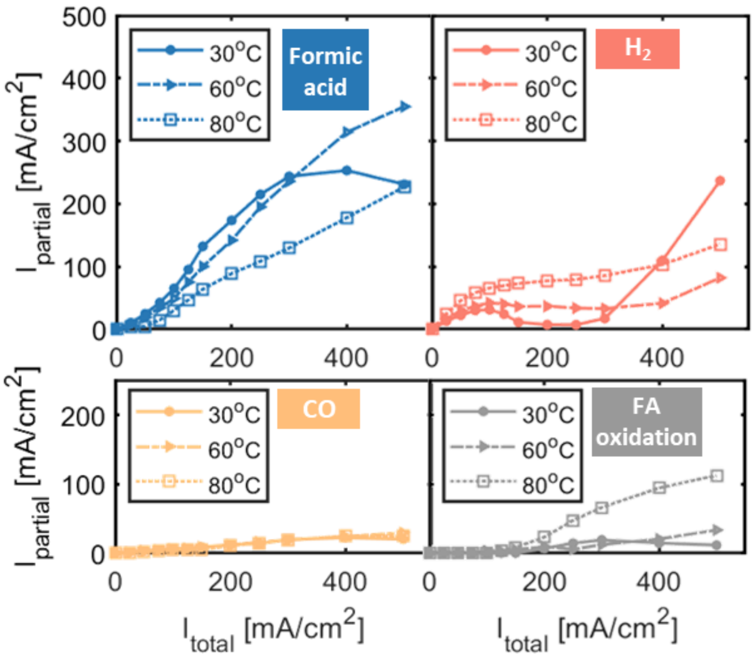


Figure S 10. Partial current densities for different reactions when the cell is operated at 30, 60, and 80 °C with Pt/C anode and 80 µm AEM.

Cell operating conditions can play a role in the transport, accumulation, and kinetics for formic acid oxidation. With a similar boiling point to water (100.8 vs 100°C at 101.3 kPa), the transport and accumulation of formic acid will be impacted by cell temperature. Figure S10. displays partial current densities as a function of temperature. As operating temperature increases, formic acid FE decreases, which is accompanied by the increased partial current density for hydrogen evolution reaction (HER). The rate for formic acid oxidation is also higher at higher temperatures, while in constrast, CO production is less sensitive to the temperature change. While we observed the net effects of varying cell temperature, the device level study highlights the need for more fundamental work in extraction of activation energies for CO2R and HOR on various catalsyts to enable more predictive rather than descriptive modeling efforts.

### Techno-economic analysis (TEA)

Our standard TEA includes four steps: (1) a detailed process flow diagram based on research data, (2) capital and project cost estimations using in-house models, (3) a

discounted cash flow economic model, and (4) the calculation of minimum selling price (MSP). The process flow diagrams include the core $CO_2$-to-FA conversion, recycling of unconverted feedstocks, and product purification stages. The analysis scale is based on $CO_2$ emissions from a 100 million gallon per year (MGY) bioethanol plant as it represents an appealing $CO_2$ source with high purity and low-cost $CO_2$. The mass flowrate of $CO_2$ ($M_{CO_2}$) input is calculated based on Eq.8, where $E_{g.f}$ is ethanol production (100MGY), $\theta_{E.f}$ is $CO_2$ emission factor (6.6lb per gallon ethanol)[2] and $O_t$ is operating hours per year (7884 h/year).

$$M_{CO_2} = \frac{\sum(E_{g.f} \cdot \theta_{E.f}) \cdot 10^6}{2.205 \cdot O_t}$$

Based on $CO_2$ mass flowrate, the materials and energy required for FA production are quantified and used to estimate capital and operating expenses. The key process and economic assumptions for TEA are summarized in Table S2. Other standard assumptions for discounted cash flow model and MSP calculation follow our published method[3].

Table S 2. Summary of major process and economic assumptions for $CO_2$-to-FA TEA

| **Process parameters** | **Value** | **Ref** |
|---|---|---|
| $CO_2$ mass flowrate (kg/h) | 37965 | [2] |
| Electrolyzer cost ($/m$^2$) | 5000 | [4] |
| Electrolyzer replacement interval | 7 | [3] |
| Electrolyzer replacement ratio | 15% | [3] |
| **Economic parameters** | | |
| State-of-the-art electricity price ($/kWh) | 0.068 | [3] |
| State-of-the-art electrolytic hydrogen price ($/kg) | 4.5 | [5] |
| Projected future electricity price ($/kWh) | 0.03 | [3] |
| Projected future electrolytic hydrogen price ($/kg) | 2.31 | [5] |
| $CO_2$ price ($/tonne) | 40 | [3] |

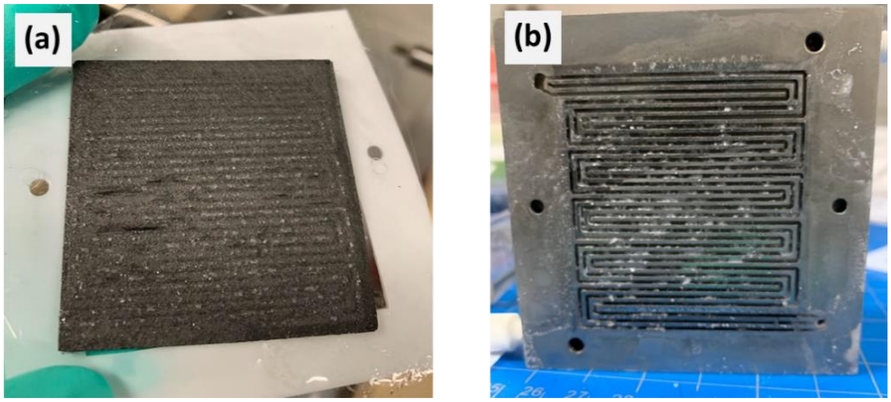


Figure S 11. Images of the (a) back of the cathode GDE and (b) flow field of the single CEM MEA after 2 hours of operation under 200 mA/cm$^2$. Significant amount of salt accumulation can be observed.

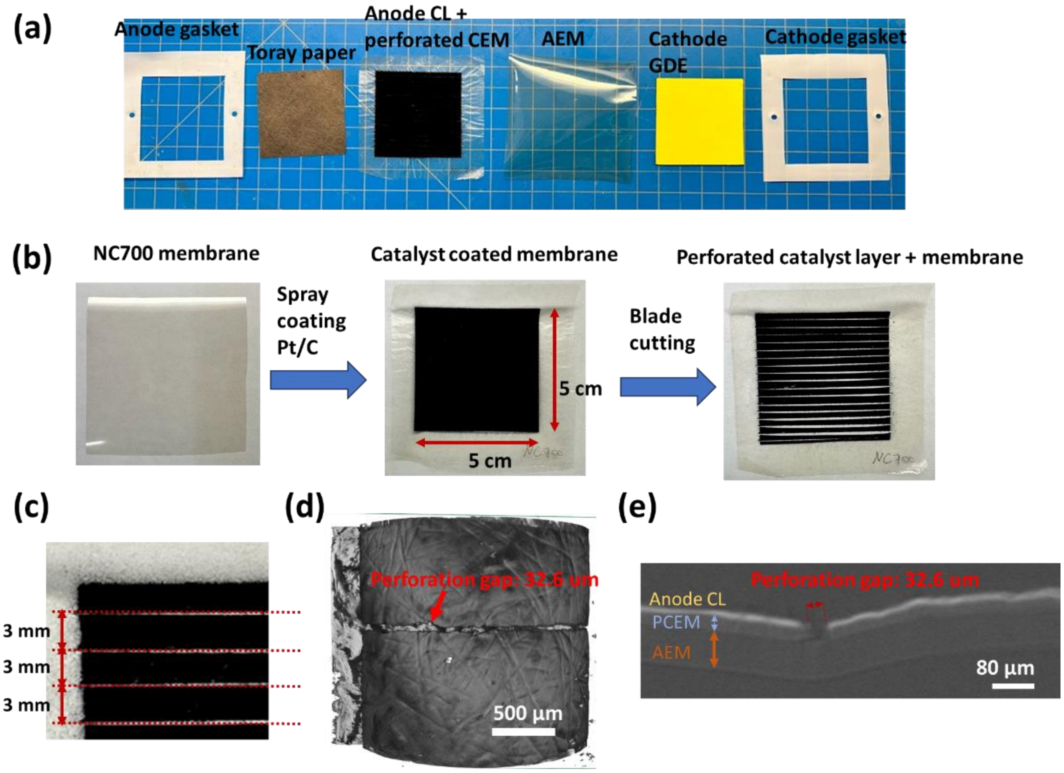


Figure S 12 (a) Details of different components for the cell assembly. From left to right are components assembled from the anode to the cathode. (b) Perforated catalyst coated cation exchange membrane preparation process. (c) Zoomed in figure of the perforation and the spacing of the parallel cutting lines. (d) 3D structure of the X-ray CT

image showing the perforated cation exchange membrane with perforation gap of 32.6 µm. (e) cross sectional image of the X-ray CT reconstruction showing the perforated cation exchange membrane with perforation gap of 32.6 µm, anode catalyst layer, and the anion exchange membrane.

## Supplementary References


1. Chen, Y. *et al.* The effect of catholyte and catalyst layer binders on CO2 electroreduction selectivity. *Chem Catalysis* **2**, 400–421 (2022).
2. Kheshgi, H. S. & Prince, R. C. Sequestration of fermentation CO2 from ethanol production. *Energy* **30**, 1865–1871 (2005).
3. Huang, Z., Grim, R. G., Schaidle, J. A. & Tao, L. The economic outlook for converting CO2 and electrons to molecules. *Energy Environ. Sci.* **14**, 3664–3678 (2021).
4. Badgett, A. *et al.* An economic analysis of the role of materials, system engineering, and performance in electrochemical carbon dioxide conversion to formate. *Journal of Cleaner Production* **351**, 131564 (2022).
5. H2A: Hydrogen Analysis Production Models. https://www.nrel.gov/hydrogen/h2a-production-models.html.
